# Identification of a flagellar protein implicated in the gravitaxis in the flagellate *Euglena gracilis*

**DOI:** 10.1038/s41598-018-26046-8

**Published:** 2018-05-15

**Authors:** Adeel Nasir, Aude Le Bail, Viktor Daiker, Janine Klima, Peter Richter, Michael Lebert

**Affiliations:** 1 Cell biology department, Friedrich Alexander University, Erlangen, Germany; 2 Biochemistry department, Friedrich Alexander University, Erlangen, Germany

## Abstract

Flagellated cells are of great evolutionary importance across animal and plant species. Unlike higher plants, flagellated cells are involved in reproduction of macro-algae as well as in early diverging land plants. *Euglena gracilis* is an emerging flagellated model organism. The current study reports that a specific calmodulin (CaM2) involved in gravitaxis of *E. gracilis* interacts with an evolutionary conserved flagellar protein, EgPCDUF4201. The subsequent molecular analysis showed clearly that EgPCDUF4201 is also involved in gravitaxis. We performed subcellular localization of CaM2 using immunoblotting and indirect immunofluorescence. By employing yeast two-hybrid screen, EgPCDUF4201 was identified as an interaction partner of CaM2. The C-terminus of EgPCDUF4201 is responsible for the interaction with CaM2. Silencing of N- and C-terminus of EgPCDUF4201 using RNAi resulted in an impaired gravitaxis. Moreover, indirect immunofluorescence assay showed that EgPCDUF4201 is a flagella associated protein. The current study specifically addressed some important questions regarding the signal transduction chain of gravitaxis in *E. gracilis*. Besides the fact that it improved the current understanding of gravity sensing mechanisms in *E. gracilis*, it also gave rise to several interesting research questions regarding the function of the domain of unknown function 4201 in flagellated cells.

## Introduction

*Euglena gracilis* is a photosynthetic, eukaryotic unicellular organism which is a member of the Euglenozoa along with the parasites of the genera *Trypanozoma* and *Leishmania*^1^. Although this phylogenetic group has evolved independently from the Archaeplastida (including the green algae), *E. gracilis* contains chloroplasts surrounded by three membranes probably acquired by secondary endosymbiosis of a green alga^2^. Additionally, in absence of a sufficient light source, *E. gracilis* can also take up nutriments heterotrophically.

*E. gracilis* responds to different environmental cues such as oxygen, light and gravity^3,4^. Among these stimuli, light and gravity are of great importance for *E. gracilis* because a balance between negative gravitaxis (directional movement away from gravity) and positive phototaxis (directional movement towards light source) facilitate cells to reach an optimal niche in the water column^5^.

Predominantly, *E. gracilis* shows a negative gravitaxis behavior. However, as shown under laboratory standard conditions cells show a transition from positive to negative gravitaxis as culture grows from young to old respectively. It has been demonstrated, in parabolic flight conditions (transition from hyper g to micro g as well as from micro g to hyper g), that this orientation is an active physiological process in which the beating of the flagella is involved and controlled by gravity^6^. The influence of microgravity conditions on gene expression has also been studied during a space craft flight^7^. However, the exact nature of the receptor responsible for gravitaxis has not yet been elucidated in *E. gracilis*. An initial inhibitor study established the involvement of stretch sensitive calcium ion channels (SSCIC) in gravitaxis^8^. Furthermore, the involvement of a putative transient receptor potential (TRP) was suggested in a follow-up study using RNA interference^9^ (RNAi). However, additional studies are required in this direction as several putative stretch sensitive/mechanosensitive channels have been identified in the mRNA library of *E. gracilis* (unpublished data). Besides this open question regarding the exact nature of gravitaxis specific SSCIC, a reasonable progress has been made regarding the underlying molecular mechanism of gravitaxis in *E. gracilis*. For instance, a current working model of gravitaxis was established based on a series of experiments^10^. The working model suggests that the SSCIC are activated as the cells deviate from their vertical swimming pathway leading to a calcium ion influx. In turn, calcium binds to a calmodulin that further activates an adenylyl cyclase. The resulting cAMP activates a protein kinase A which is suggested to modulate the functions of various proteins in the flagella. Recently, silencing of a specific protein kinase A (PKA) through RNAi elucidated its involvement in gravitaxis as well as in phototaxis^11^. In another study, silencing the specific calmodulin 2 protein (CaM2) resulted only in impaired gravitaxis in *E. gracilis*^12^.

This report provides information about the subcellular localization of CaM2 as well as the identification of an interaction partner employing yeast two-hybrid screens. The resulting binding partner was further characterized by heterologous expression, *in silico* analysis and its subcellular localization was analyzed.

## Results

### CaM2 is present in the cell body and the flagella of *E. gracilis*

In order to better understand the function of CaM2, we aimed to identify its subcellular localization by indirect immunofluorescence (IIF) and cell fractionation followed by Western blot, using anti CaM2 genomic antibody. Prior to the immuno-localization experiments, the specificity of the commercially raised genomic anti-CaM2 antibody was determined. Genomic antibodies facilitate the detection of an antigen in its denatured and native conformation, were used. Their reactivity to the *E. gracilis* protein lysate (Supplementary Figure 1a) and to CaM2 fused to the Glutathion S Transferase (GST, Supplementary Figure 1b) was checked under denaturing conditions by Western blot. Both experiments showed a single band at the expected size. The specificity was further confirmed by generating a knockdown mutant (Supplementary Figure 1c). The protein lysates from the wild type and the mutant were analyzed by Western blot with the anti-CaM2 antibody. Whereas the control with an anti-tubulin antibody showed an equal amount of proteins in the two samples, no protein could be detected in the knockdown mutant with the anti-CaM2 antibody. Altogether, this data confirmed the specificity of the anti-CaM2 antibody.

**Figure 1:**
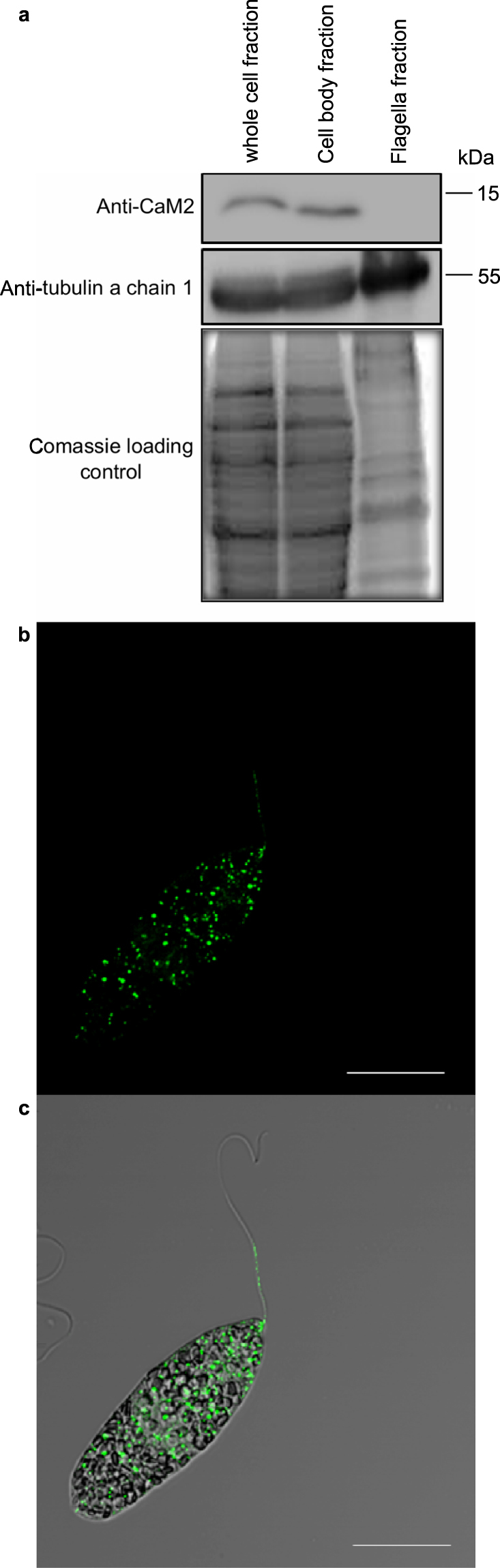
CaM2 resides in the cell body of *E. gracilis*. (**a**) Western blots of the cell fractions with CaM2 antibody (upper panel) and tubulin antibody (middle panel). (**b**,**c**) Indirect immuno-fluorescent assay with anti-CaM2 antibody. Scale bar = 20 µm.

**Figure 2:**
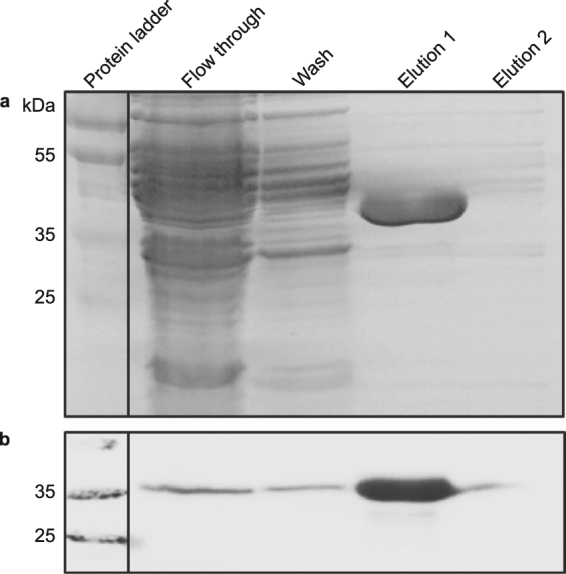
Heterologous expression and purification of GST-CaM2. (**a**) Coomassie stained SDS polyacrylamide gel of the non-purified (flow through and wash) and the purified (elutions) GST (left) and GST-CaM2 (right). (**b**) Western blot detected with a monoclonal GST antibody.

The determination of CaM2 subcellular localization was carried out by cell fractionation studies followed by Western blot and by IIF assay. The separation of the cell body and the flagella fraction was performed and the purity was confirmed microscopically (Supplementary Figure 2). The quantitative Western blot clearly showed that CaM2 was abundant in the cell body fraction, whereas no visible signal of CaM2 could be observed in the flagella fraction (Fig. 1a). In addition, IIF assay showed that CaM2 is scattered all over the cell body in a spotted pattern, but a weak signal was also visible in the flagellum (Fig. 1b,c). To validate the specificity of the signal, a CaM2 knockdown mutant cell culture of *E. gracilis* was generated. In both wild type and knockdown mutant cells, the signal appeared as spots (Supplementary Figure 3). However, quantitative analysis revealed that the number of spots decreased significantly in the knockdown mutant cells, which proved that the spotted pattern represented a specific signal (Supplementary Figure 3c). Although CaMs are generally soluble proteins, CaM2 appeared as aggregated spots. To determine if CaM2 was a cytoplasmic protein, a GST-CaM2 fusion protein was generated and expressed in *E. coli*. The soluble fraction of the prepared crude protein lysate GST-CaM2 transformed cells of *E. coli* was used, and the fusion protein was purified. The expression of GST-CaM2 from the soluble fraction of the cell lysate indicates that GST-CaM2 is expressed as a soluble protein in *E. coli* (Fig. 2).

### CaM2 interacts with a protein of unknown function

**Figure 3:**
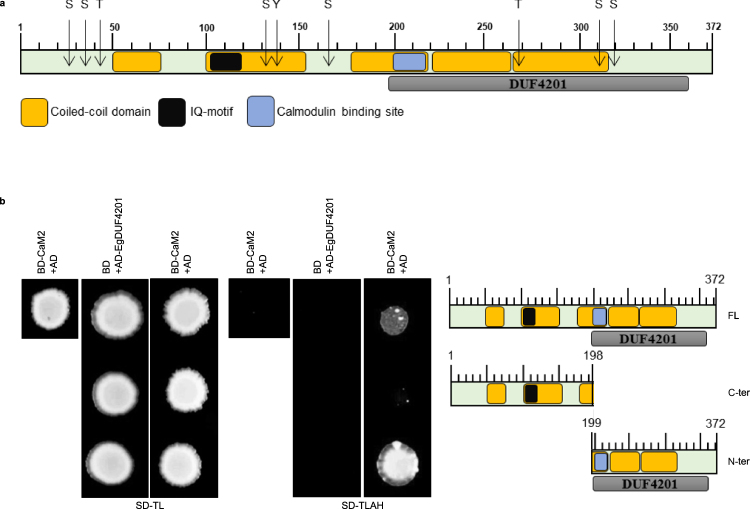
EgPCDUF4201 interacts with CaM2. (**a**) Protein domains of EgPCDUF4201. (**b**) Yeast two-hybrid assay between CaM2 and truncations of EgPCDUF4201. BD = binding domain. AD = activation domain. FL = full length. C-ter = C-terminus. N-ter = N-terminus. SD-TL (Synthetic Define Medium without Leucine and Tryptophane). SD-TLAH (Synthetic Define Medium without Leucine, Tryptophane, Adenine and Histidine).

To further understand the role of CaM2 in the gravitaxis of *E. gracilis*, a yeast two-hybrid screen was performed to find its putative interacting partners using a normalized commercially raised cDNA library of *E. gracilis*. Four clones identified in the screen were positive in the retransformation assay. Among them, a protein containing the domain of unknown function (DUF) 4201 was found and named EgPCDUF4201. The 5′and 3′ RACE PCR was performed to obtain the full length cDNA sequence of *EgPCDUF4201*. The obtained full length cDNA sequence of *EgPCDUF4201* encoded a protein of 372 amino acids. An *in silico* analysis run to identify potential additional conserved domains showed that, in addition to the DUF4201 domain, 5 coiled-coil domains, a predicted IQ motif (104–123 residues) and a putative calmodulin binding site (202–221 residues) were present (Fig. 3a). The search for potential phosphorylation sites revealed 6 serine, 2 threonine and 1 tyrosine phosphorylation sites at various positions in the sequence of EgPCDUF4201 (Fig. 3a). To determine more precisely the region interacting with CaM2, the interaction of the full length CaM2 bait protein was tested with full length (1–372 amino acids), N-terminus truncation (1–198 amino acid), and C-terminus truncation (199–372 amino acid) of EgPCDUF4201. The results indicated that the C-terminal domain (199–372 amino acids) of EgPCDUF4201 interacts with CaM2, whereas the N-terminal domain does not (Fig. 3b). Moreover, the full length EgPCDUF4201 was also able to interact with CaM2, but at a weaker extent (Fig. 3b).

### Functional analysis of putative interaction partner of CaM2

**Figure 4:**
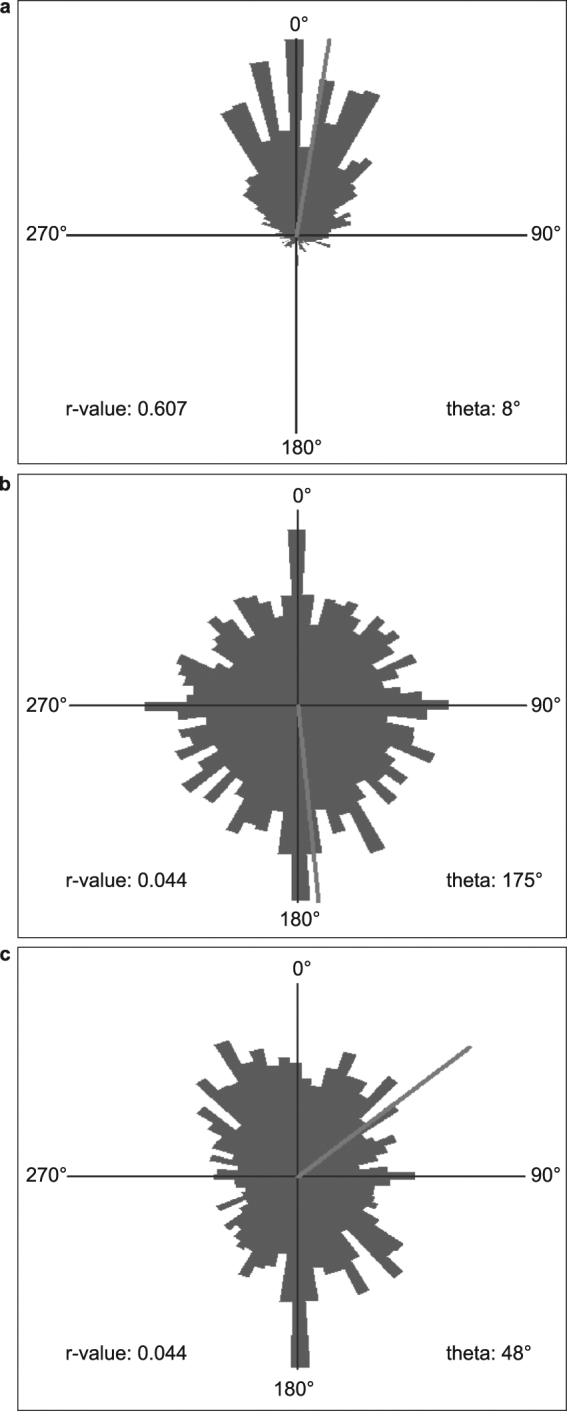
EgPCDUF4201 is implicated in the gravitaxis of *Euglena gracilis*. Representative histograms of the directional movements of wild type (**a**) and *EgPCDUF4201* C-terminal targeted (**b**) and N-terminal targeted (**c**) knockdown mutants. The size of the angular sectors (represented in black) reflects the number of cells moving in the corresponding direction.

In order to understand the function of the identified CaM2 putative interaction partner, silencing of *EgPCDUF4201* was carried out by employing RNAi. Two regions of the genes were targeted, the first one in the N-terminal region, the second one in the DUF4201 domain. The down-regulation of the gene was confirmed by real time PCR (Supplementary Figure 4). Both knockdown mutants of *EgPCDUF4201* showed an impaired gravitaxis with random orientation of their swimming pattern, similarly to the *CaM2* knockdown mutants^12^ and contrarily to the wild type where the majority of the cells are swimming upward (Fig. 4, Supplementary Figure 6). However, the swimming speed of the mutants was not altered (Supplemental Table 1), which can rule out a defective flagella formation. These findings provided the evidence that EgPCDUF4201 is involved in gravitaxis of *E. gracilis* and shares a functional association with CaM2.

### EgPCDUF4201 is a soluble flagellar protein

**Figure 5:**
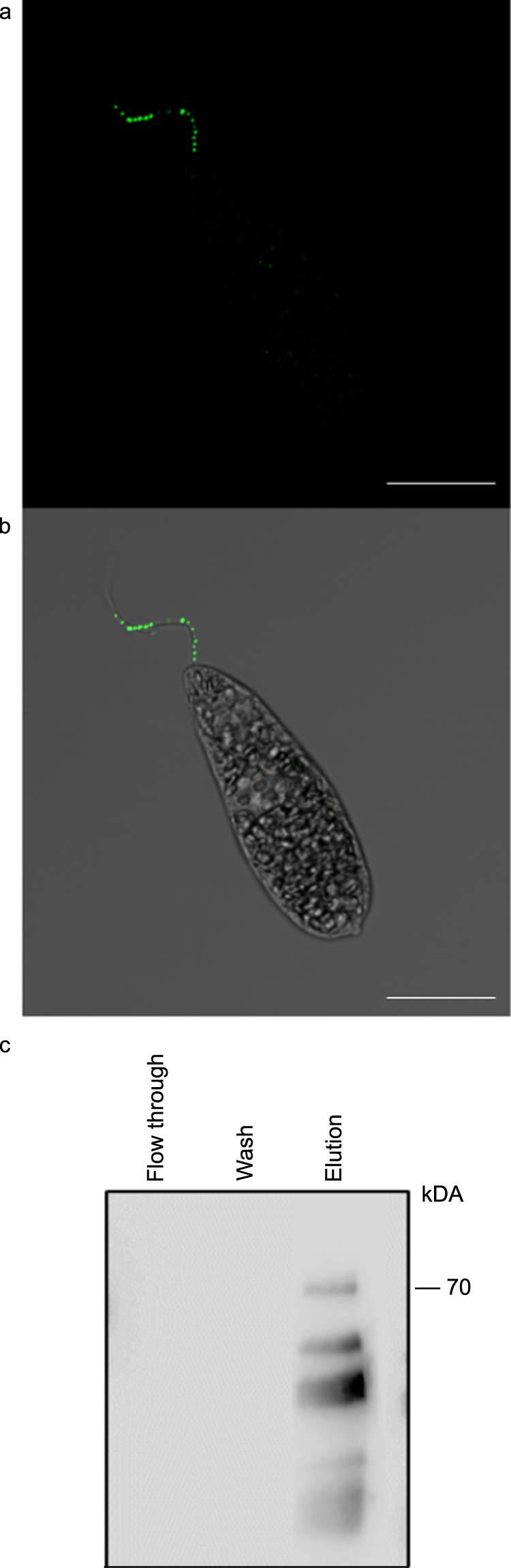
EgPCDUF4201 is a soluble protein localized in the flagella of *E. gracilis*. (**a**,**b**) Indirect immuno-fluorescent assay with antiEgPCDUF4201 immune serum. (**c**) Western blot of the fusion GST- EgPCDUF4201 with a GST antibody.

A custom made polyclonal mouse antiserum raised using the full length EgPCDUF4201 protein was used to immunolocalize EgPCDUF4201 at the subcellular level. The IIF assay clearly indicated that EgPCDUF4201 is present in the flagella of *E. gracilis*, from the base to the apex, in a dotted pattern (Fig. 5a,b), whereas no immune reactivity was observed from the pre-immune serum (Supplemental Figure 5a). To further check this signal, a *EgPCDUF4201* knockdown mutant culture was raised. A decrease in the fluorescence intensity and of the number of spots was observed in the knockdown mutant compared to the wild type, confirming the specificity of the anti-EgPCDUF4201 antiserum (Supplementary Figure 5a). In addition, the flagella length of the knockdown mutant was also determined to understand their putative involvement in the structure of the flagella. No statistical significant difference was observed in the flagella length of *EgPCDUF4201* knockdown mutant cells compared to the wild type cells (Supplementary Figure 5c). Finally, the localization of EgPCDUF4201 is not changed in the *CaM2* knockdown mutant (Supplementary Figure 6).

In addition, in order to identify if the protein was membrane associated or soluble, a fusion protein GST-EgPCDUF4201 was expressed in *E. coli* and a Western blot was performed with the soluble fraction. The anti-GST antibody detected the fusion protein, revealing that EgPCDUF4201 is expressed as a soluble protein in *E. coli* (Fig. 5c).

### The DUF 4201 is present in a wide range of organisms

A BLAST search has been performed in order to find which organisms possess proteins with the DUF 4201. In addition to being present in the closely related organisms *Trypanosoma* and *Leishmania*, this domain was found in a very wide range of species. In algae and plants, this domain is present in the available genomes of flagellated alga (*eg Chlamydomonas*), of the brown alga *Ectocarpus siliculosus*, whose gametes and spores are flagellated, and of the early diverging land plants which use sperm cells to reproduce (*eg* the bryophytes *Physcomitrella* and *Marchantia* and the lycophyte *Sellaginella*), but was not found in the angiosperm genomes. This domain was not found exclusively in plant genomes but is also present in fungus and animals. These data showed that the DUF 4201 domain is extremely conserved across the phylogenetic groups which possess at one point of their life cycle flagellated cells.

## Discussion

A previous report revealed the involvement of a specific calmodulin, CaM2, in the gravity signal transduction chain of *E. gracilis* resulting in gravitaxis^12^. The current study provides a detailed account regarding the subcellular localization of CaM2 and identifies a new factor implicated in the gravitaxis.

The cell fractionation study indicated that CaM2 resides in the cell body of *E. gracilis* but no signal could be detected on the flagella fraction. However, the IIF assays indicated that CaM2 is abundantly present in the cell body, and in a weak extend also in the flagella. Furthermore, both endogenous and heterologous CaM2 proteins (GST-CaM2) were detected in the soluble fraction of the cell lysate. However, IIF assay shows that the protein aggregates and appears as spots. Similar pattern has also been reported for other soluble proteins. For instance, in neuronal cells, a CaM dependent protein kinase II (CaMKII) was shown to self-aggregate near the endoplasmic reticulum under certain stress conditions^13^. Therefore, in *E. gracilis* as in neuronal cells, a cellular stress before cell fixation can result in CaM2 aggregation in the cell body. Moreover, it has been recently shown that at high Ca^2+^ level, which can be caused by cellular stresses, CaMs are bunched in well-defined regions, suggesting their association with cellular complexes^14^. On the contrary, at low Ca^2+^ concentrations, CaMs are very motile proteins that are quickly available to propagate signals^14^. Even if the fixation could have slightly altered the CaM2 localization, CaM2 was detected all around the cell body, which is in accordance with the current working model of gravitaxis^10^. Indeed, the calcium ions coming from the influx caused by the activation of SSCIC binds to CaM2. CaM2 being one of the primary actors of the signalization chain, it has to be quickly accessible at every sides of the cell, since the calcium influx could arise at any part of the plasma membrane, depending of the direction of the swimming deviation.

The cells deviating from their upward swimming pattern have to be reoriented in order to stay in the best suitable place in the water column. This reorientation is achieved by a change in the beating of the flagella. Very interestingly, we identified a newly putative interaction partner of CaM2, EgPCDUF4201, localized in the flagella. The interaction occurred specifically with the C-terminus of the protein which contains the DUF4201. One part of this domain possesses also a putative calmodulin binding site, suggesting that the interaction could occur more specifically on this portion of the protein and further strengthens the results of the yeast two-hybrid. The IIF revealed that EgPCDUF4201 is distributed following a spotted pattern all along the flagellum, including at the base. We suggest that this protein is an intermediate factor between CaM2 and the dynein proteins which make the molecular motive force necessary for the flagella beat^15^.

EgPCDUF4201 contains the DUF 4201. This domain is characterized as a coiled-coil domain and is present only in the ciliated and flagellated cells. The coiled-coil containing domain proteins are reported to function as skeletal proteins, motor proteins, pH-dependent coiled-coil switches and molecular recognition systems which make them a group of multifaceted proteins^16^. There are few reports regarding DUF4201 containing proteins. Notably, two DUF4201 containing proteins of *Homo sapiens*, CCDC113 and CCDC96, are involved in cilia formation of sperm cells and have been reported to be localized in the centriolar satellites and centrosome^17^. These structures are essential for the nucleation of the axonemes of the flagella. Although CCDC113 and CCDC96 share 30% and 38% similarity (amino acids) with EgPCDUF4201, respectively, their role is likely different from EgPCDUF4201. This latter is located in the flagella but is not implicated in their formation, as its down-regulation does not alter the flagella length.

Interestingly, two homologues of EgPCDUF4201, termed flagella associated protein FAP 184 and FAP 263 are present in *Chlamydomonas* and reported as phosphoproteins^18^. The bioinformatics analysis also predicted EgPCDUF4201 to be a phosphoprotein. Hence, it is plausible that post-translational modifications of EgPCDUF4201 through phosphorylation plays a role in the modulation of its function. Moreover, the current model of the gravity signal transduction chain of *E. gracilis* includes a kinase, PKA^11^. We can therefore speculate that PKA could phosphorylate EgPCDUF4201. In any case, it strengthens the fact that phosphorylation plays a role in the transduction chain of gravitaxis in *E. gracilis*.

It has been shown previously, in micro- and hyper-gravity experiments, that the beating of the flagella was an active process controlled by gravity^6^, but no specific flagellar protein playing a role in gravitaxis had yet been identified. The identification of EgPCDUF4201 brings thus a new major factor in the current working model, and is the first step to explain the change of the flagellar beat to reorient the cells. However, the mode of action of EgPCDUF4201 has still to be explored. Finding the interaction partners will help to understand if the protein acts on the beating of the flagella by interacting directly with the dyneins, or is only an intermediary actor of the signaling pathway leading to a proper beating pattern.

## Conclusion

The protein CaM2 previously identified as a member of the gravity signal transduction chain of *E. gracilis* is located in the cell body and in the flagella in a spotted pattern. Furthermore, CaM2 interacts with EgPCDUF4201 which is localized in the flagellum. The down regulation of the protein leads to an impaired gravitaxis. Thus, the study demonstrated the role of a novel protein in the gravity signal transduction chain in *E. gracilis*. This novel protein presents homologues in the other organisms possessing cilia or flagella, but their functions are still largely unknown and most likely not related to gravitaxis only.

## Materials and Methods

### Cell culture and growth conditions

The experimental organism*, Euglena gracilis* KLEBS, strain Z, was obtained from the algal culture collection at the University of Göttingen, Germany^19^. All experiments were performed with axenic cell cultures grown in an organic medium^20^. The cultures were grown at 20 °C under continuous light with an intensity of 54 μmol/(m^−2^ s^−1^) from mixed cool white and warm tone fluorescent lamps.

### Motion analysis

The motion analysis was performed using a cell tracking software, Wintrack 2000^21^ coupled with a custom made device (Manual Ecotox) which consists of a microscope with a horizontal beam path and a CCD camera to observe cell movement with respect to the direction of the gravity vector. A blue light LED provides the phototactic stimulus, and an infrared LED (λ = 875 nm) allows for observation without influencing the cells. The cells were transferred to a custom made disposable glass cuvette (two plain glass slides stuck together with a U-shaped double adhesive tape, with a space in between to pour the cell suspension) and positioned vertically in the horizontal microscope to determine the gravitactic orientation. The incoming video images were analyzed by the Wintrack 2000 software in order to determine the movement vectors, various physiological (cell motility, cell shape, velocity, and area of the objects etc.) and statistical parameters of the swimming cells simultaneously. Precision of orientation (r-value) was determined as described earlier^22^. The r-value ranges between 0 (when all cells swim randomly) and 1 (when all cells swim in a single direction). The movement of the cells under observation is represented as a circular histogram with 64 angular sectors^23^. The upward direction in the histogram (0°) corresponds to an upward direction of the cells. The length of the angular sectors reflects the number of cells moving in the corresponding direction.

### Molecular and genetic analysis

The total RNA was extracted using the Trizol reagent (Invitrogen, Cat.No. 15596-026) and its concentration was estimated spectrophotometrically, using a nano-drop photometer (Thermoscientific, USA). The knockdown mutant cell culture was evaluated by quantification of mRNA expression levels by real time PCR. The equal amount of total RNA was transcribed into cDNA using QuantiTECT reverse transcription kit (Qiagen). The quantification of the mRNA levels was carried out in the CFX96 TouchTM Real-Time PCR detection system using QuantiTect SYBR Green PCR Kits (Qiagen, annealing/extension for 30 seconds at 60 °C, 40 cycles). The cDNA was diluted (1:3 or 1:500) with deionized water before PCR and the gene specific primers (listed in the Supplementary Table 2) were used for amplification, whereas actin was used as reference gene.

For gene silencing studies, a RNAi approach was adopted. The targeted gene sequence was amplified (primers listed in the Supplementary Table 2) using Taq polymerase. The amplified PCR product was ligated to a custom synthesized T7 promoter using T4 DNA ligase enzyme (New England BioLabs). The T7 promoter conjugated PCR product was used to generate dsRNA using the MEGAscript® RNAi Kit (Ambion). Subsequently, approximately 10^6^ cells per 100 μL of organic medium were transformed by electroporation with 15–20 μg of dsRNA to generate knockdown mutant cells, whereas control cells were electroporated with elution buffer only. The electroporation was carried out as described earlier^11,24,25^.

For performing RACE PCR, the total RNA was extracted as described above and cDNA synthesis was carried out using the Mint2 cDNA synthesis kit (Evrogen) as described by Frohman^26^. A set of gene specific sense and anti-sense primers (listed in Supplementary Table 2) were designed to target the known sequence of the gene at various lengths. In order to perform 5′-RACE PCR, gene specific anti-sense primers were used in conjunction with a custom design cap primer which anchor the trans-splicing 5′ cap structure found in most mRNA of *E. gracilis* as described by Tessier *et al*.^27^, whereas 3′-RACE PCR was performed by using gene specific sense primers and custom designed oligo-dT primer with two degenerated nucleotides. Two commercially available kits (RACE kit 2nd generation from Roche and FirstChoice® RLM-RACE Kit from Ambion) were used.

### Expression of plasmid constructs

Full length coding region of *CaM2* (accession# EU935858) was amplified and the restricted digested product was ligated into pGBKT7 (Clonetech) to be expressed under the T7 promoter. Similarly, the full length coding region of the *CaM2* and the *EgPCDUF4201* was amplified and ligated into pCR™II-Blunt-TOPO® (Invitrogen). Subsequently, the inserts were restricted digested and sub-cloned into pGEX4T2 (GE Healthcare) to perform GST-fusion expression of protein under TAC promoter. Additionally, two truncated (1–594 and 595–1119) and a full length coding region of *EgPCDUF4201* with a stop codon was amplified and ligated first into pCR™II-Blunt-TOPO® and sub-cloned into pGADHA (Dualsystemsbiotech) for expression under ADH1 promoter. The amplification of respective inserts was carried out using a proof reading DNA polymerase, Phusion (Thermo scientific).

### Construction of cDNA library of *E. gracilis* and Yeast two-hybrid screen

To determine the interaction partner of CaM2 a yeast two-hybrid screen was performed using a normalized cDNA library of *E. gracilis*. To raise a cDNA library, the RNA was extracted from *E. gracilis* cells exposed to various chemical and physical stresses (heavy metals, ultraviolent light, high light, cell culture at various ages, in microgravity, on a clinostat etc.). The extracted RNA samples were pooled and provided to a commercial vendor (LGC Genomics GmbH, Berlin, Germany) for construction of the cDNA library. The normalized cDNA library was ligated into an activation domain (AD) containing plasmid, pGAD-HA (Mobitech). The *Saccharomyces cerevisiae* strain PJ692a^28^ was transformed with the bait plasmid, pGBKT7-CaM2 using polyethylene glycol (PEG)/lithium acetate/single stranded carrier DNA method as described earlier^29^. Subsequently, the bait transformant yeast cell culture was re-transformed with 50 µg of the cDNA library by employing a library scale transformation protocol as described in MatchMaker GAL4 Two-Hybrid System user manual (Clonetech). The transformation efficiency of yeast cells was determined on SD-TL plates, whereas the interaction between two proteins was determined on SD-TLAH selection medium. Similarly, the interaction between two selected proteins was determined by observing the growth of yeast cells on SD-TLAH selection medium. The auto-activation of bait or prey protein was determined by observing the growth of yeast cells co-transformed with empty plasmids.

### Antibodies

The production of custom antibodies against CaM2 was outsourced to a commercial firm, SDIX, USA. The antibody was raised using a genomic antibody approach against 91 amino acids (58–148) of CaM2^30^. Similarly, the production of crude mouse antiserum against *E. gracilis* protein containing the domain of unknown function (EgPCDUF4201) was outsourced to a commercial firm, Eurogenetec, Belgium. The antiserum against EgPCDUF4201 was raised using genetic DNA immunization method against the whole protein. Anti-rabbit tubulin alpha chain antibody (Catalogue#AS10 680, Agrisera, Sweden), and anti-mouse anti-Glutathione-S-Transferase (GST) (Sigma Aldrich, USA) antibody were also used. And commercially available anti-Rabbit IgG-Peroxidase antibody (Sigma Aldrich, USA), Anti-Mouse IgG-peroxidase antibody (Sigma Aldrich, USA), and Alexa Fluor® 488-Anti-Rabbit IgG (H + L) (Jackson immune research laboratories, USA) were used as secondary antibodies in this study.

### Protein sample preparation from *E. gracilis*

The cells were harvested by centrifugation (11000 × g, 10 min) and washed twice with deionized water. The cell pellet was suspended in lysis buffer (40 mM Tris-HCl, pH 8) supplemented with a protease and phosphatase inhibitor cocktail (10 μL mL^−1^, product number 78443, Thermoscientific). The cell disruption was carried out with an ultra-sonic homogenizer (Bandelin ultrasonic converter, Sonoplus hd UW 2070 and HF generator, GM 2070, Germany) by giving three pulses of 20 s at 35% intensity of total power. The samples were kept on ice throughout the process in a cold chamber (4 °C). The resultant crude lysate was resuspended in 100% acetone (three times the volume of the cell lysate) and protein precipitation was carried out at −20 °C overnight. The precipitated protein fraction was pelleted down by centrifugation (24000 g, 4 °C, 1 h) and washed twice with 80% acetone. The air dried protein pellet was suspended in 100 μL of resuspension buffer (7 M urea, 2 M thiourea, 2% CHAPS, 40 mM DTT) and transferred to a 1.5 mL microfuge tube and protein solubilization was carried out for 1 h (30 °C, 1400 cycles/min) using a heating shaker (Thermomixer comfort, Eppendorf, USA). Subsequently, the solubilized protein fraction was collected through centrifugation (20000 g, 5 min, room temperature). The concentration of protein samples was determined by Bradford assay^31^.

### SDS PAGE and immunoblotting

The SDS-PAGE was performed as described by Laemmli^32^. The separation of protein samples was carried out with 12% SDS polyacrylamide gel. The SDS polyacrylamide gels were stained using Coomassie brilliant blue R250 solution for analysis as described earlier^33^ for visual observation. For immunoblotting the protein samples from the SDS-PAGE were transferred onto a polyvinylidene fluoride (PVDF) membrane (Roti®-Fluoro PVDF, pore size 0.2 μM, Carl Roth, Germany) using Trans-Blot SD Semi Dry Transfer Cell (Bio-Rad, USA). Subsequently, the membrane was soaked in a blocking buffer, 5% nonfat dry milk in tris-buffered saline (W/V) with 0.5% tween 20 (V/V) (TBS-T) for 1 h at room temperature with a constant shaking (20 cycles/min). Afterwards, the membrane was probed with the respective primary antibody in a blocking buffer (30 cycles/min), followed by three washes with TBS-T buffer for 10 min and incubated with blocking buffer containing the respective secondary antibody. After three washes with TBS-T buffer, blots were visualized by a commercially available enhanced chemiluminescence (ECL) detection kit (PerkinElmer, USA) and the images were acquired by FUSION FX7™ Advance Chemiluminescence System (Peqlab, Germany).

### Organelle fractionation

The separation of flagella and the cell body was carried out by employing the method described in^34^ using 15 L of late logarithmic cell culture. The method was originally devised for the isolation of flagella associated with the paraflagellar body (PFB). The resultant separated flagella and the cell body fractions were collected for protein extraction.

### Indirect-Immunofluorescence confocal microscopy

For indirect-immunofluorescence (IIF) assay, samples were prepared as described earlier^35^ and cells were analyzed using a fluorophore (Alexa 488) conjugated secondary antibody. Confocal images were generating using a TCS SP2 (Leica) laser scanning microscope equipped with a HC PL APO 20× water immersion lens. Alexa 488 was excited at 495 nm and imaged between 510 and 580 nm. For the comparison of the control and the knockdown mutants, the parameters of the image acquisition were kept identical.

### Over expression of GST-fused proteins and purification

The electro-competent cells of *E. coli* strain BL21 (DE3) pLysS (Invitrogen) were transformed with expression plasmids, pGEX4T2-*CaM2* and pGEX4T2-*EgPCDUF4201*. The positive transformants were selected on LB solid medium containing 1.5% (W/V) agar and 100 µg.mL^−1^ of ampicillin. Subsequently, a single transformant colony was suspended in 5 mL of LB broth containing ampicillin (100 µg.mL^−1^) to grow overnight (37 °C, 200 cycles per min). The following day, 1000 mL of LB medium were inoculated with the overnight transformant culture of pGEX4T2-*CaM2* and pGEX4T2-*EgPCDUF4201*, respectively. Afterwards, the protein expression was induced by adding isopropylthio-β-D-galactopyranoside (IPTG) to a final concentration of 1 mM as the growing cultures reached to a certain cell density (OD600 of 0.6–0.7). The cells were collected after 5 h growth (37 °C, 200 cycles per min) by centrifugation (6000 g, 4 °C, 10 min). Following two washes with deionized water, the cell pellet was dissolved in TIF buffer (20 mM Tris, pH 8.0, 150 mM NaCl, 1 mM MgCl_2_, 0.1% NP40, 10% glycerol) with an already mixed tablet of a protease inhibitor cocktail (Complete protease inhibitor cocktail, Roche). Subsequently, cells were sonicated by giving three pulses of 20 s at 30% power using an ultra-sonic homogenizer (Bandelin ultrasonic converter, Sonoplus hd UW 2070 and HF generator, GM 2070, Germany). The soluble fraction (supernatant) was collected by centrifugation (7000 g, 4 °C, 10 min). The purification of GST fusion proteins from the soluble fraction was performed using Portino® GST columns (Macherey-Nagel, Germany) with a bed volume of 1 mL. The manual procedure of purification was adopted using a 2 mL syringe with the help of a Portino® inlet-luer adaptor (Macherey-Nagel, Germany).

### *In silico* analysis

The analysis of the predicted domains of EgPCDUF4201 was performed using expasy online tool, COILS (http://embnet.vital-it.ch/software/COILS_form.html) and an online database for the prediction of Calmodulin binding sites (http://calcium.uhnres.utoronto.ca/ctdb/ctdb/home.html). The prediction of phosphorylation sites in EgPCDUF4201 was done by using NetPhos 2.0 server (http://www.cbs.dtu.dk/services/NetPhos-2.0/).

## Electronic supplementary material

Supplementary information
