# Supplementary material for: Identification of a flagellar protein implicated in the gravitaxis in the flagellate Euglena gracilis
Source: Sci Rep. 2018 May 15;8:7605. doi: 10.1038/s41598-018-26046-8 (PMC5954063; doi:10.1038/s41598-018-26046-8)
Supplement: Supplementary file 1 — Supplementary information [file 41598_2018_26046_MOESM1_ESM.pdf]

Identification of a flagellar protein implicated in the gravitaxis in the flagellate *Euglena gracilis.*

Adeel Nasir, Aude Le Bail, Viktor Daiker, Janine Klima, Peter Richter and Michael Lebert

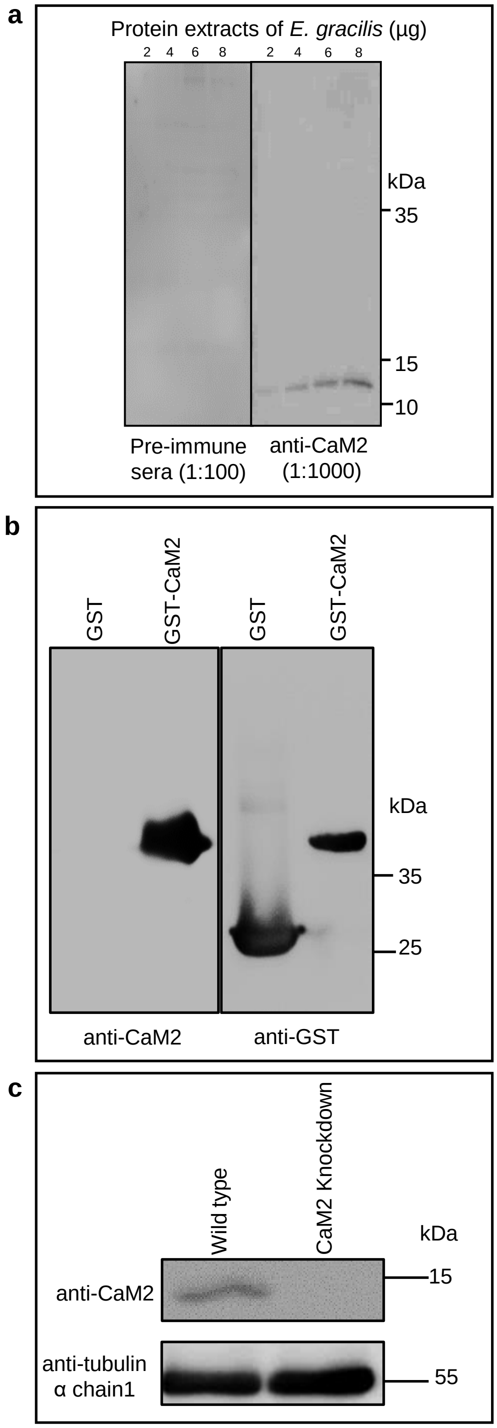

Supplementary Figure 1. Anti-CaM2 antibody characterization and validation of specificity. **a**. Western blot with different amounts of protein extracts. **b**. Western blot of the purified GST-CaM2 fusion protein. **c**. Western blot with the protein fraction of wild type cells and the CaM2 knockdown mutant cells.

a

b

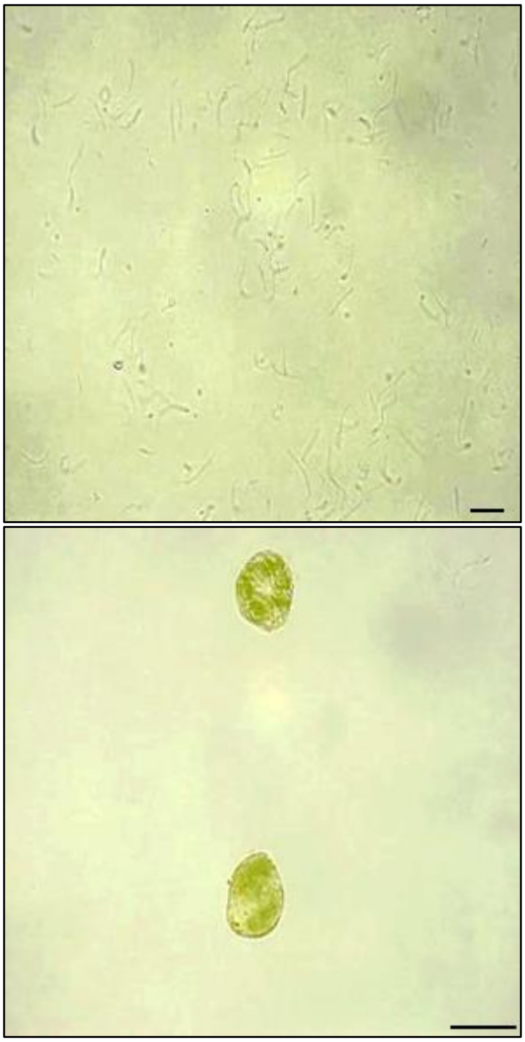

Supplementary Figure 2. Organelle fractionation. **a**. Isolated flagella fraction. **b**. De-flagellated cell bodies. Scale bar: 20 µm.

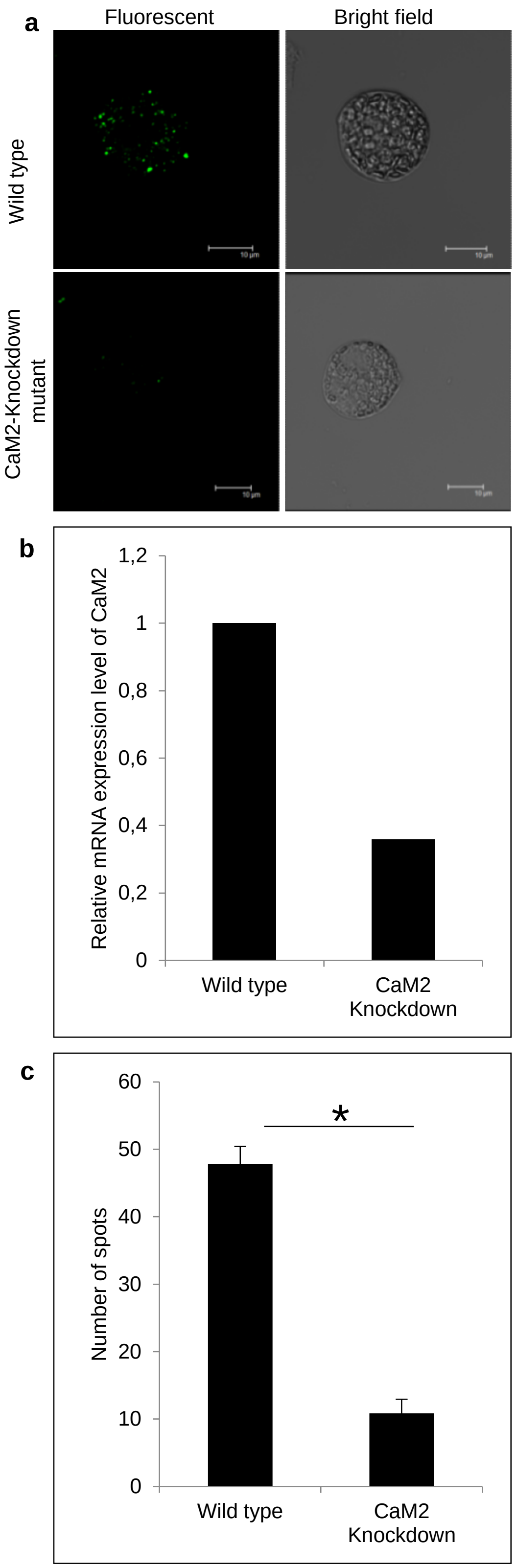

Supplementary Figure 3. Confirmation of the CaM2 localization. **a**. Indirect immuno-fluorescent assay with anti-CaM2 antibody. **b**. Expression level of CaM2 measured by real time PCR. **c**. Number of spots in wild type cells and CaM2 knockdown cells. N=50. * represents a significant difference of the student test.

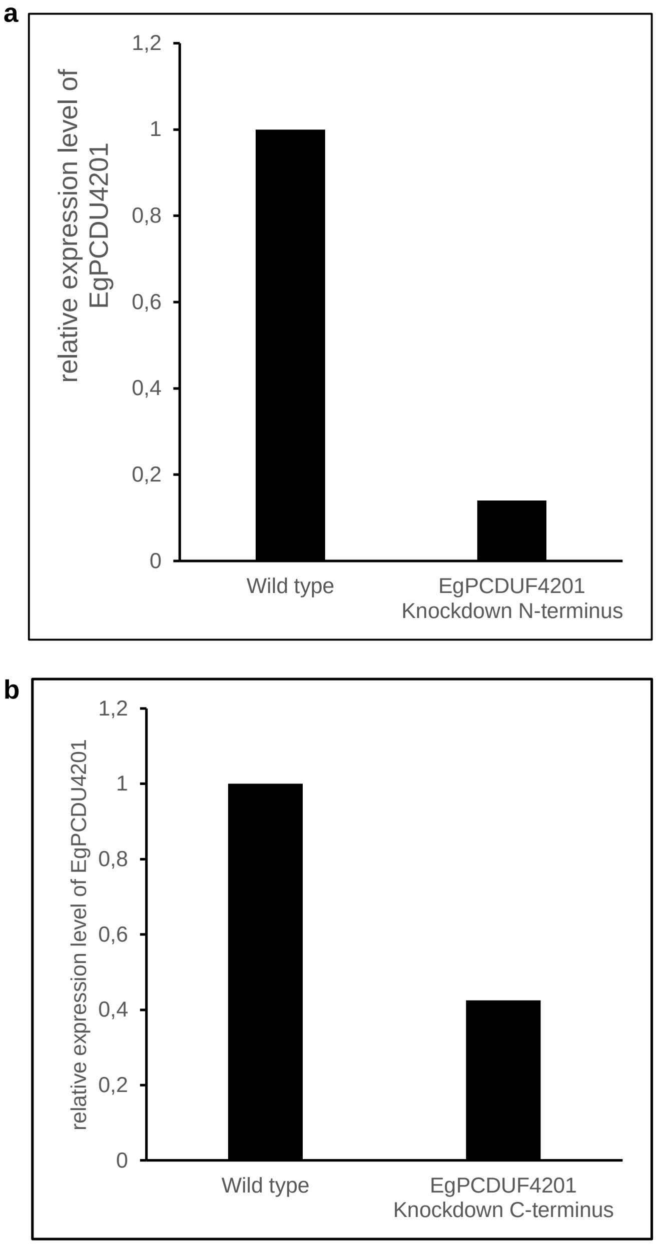

Supplementary Figure 4. Expression level of Eg*PCDUF4201*. Expression of Eg*PCDUF4201* measured by real time PCR in the N-terminus (**a**) and C-terminus (**b**) knockdown mutants.

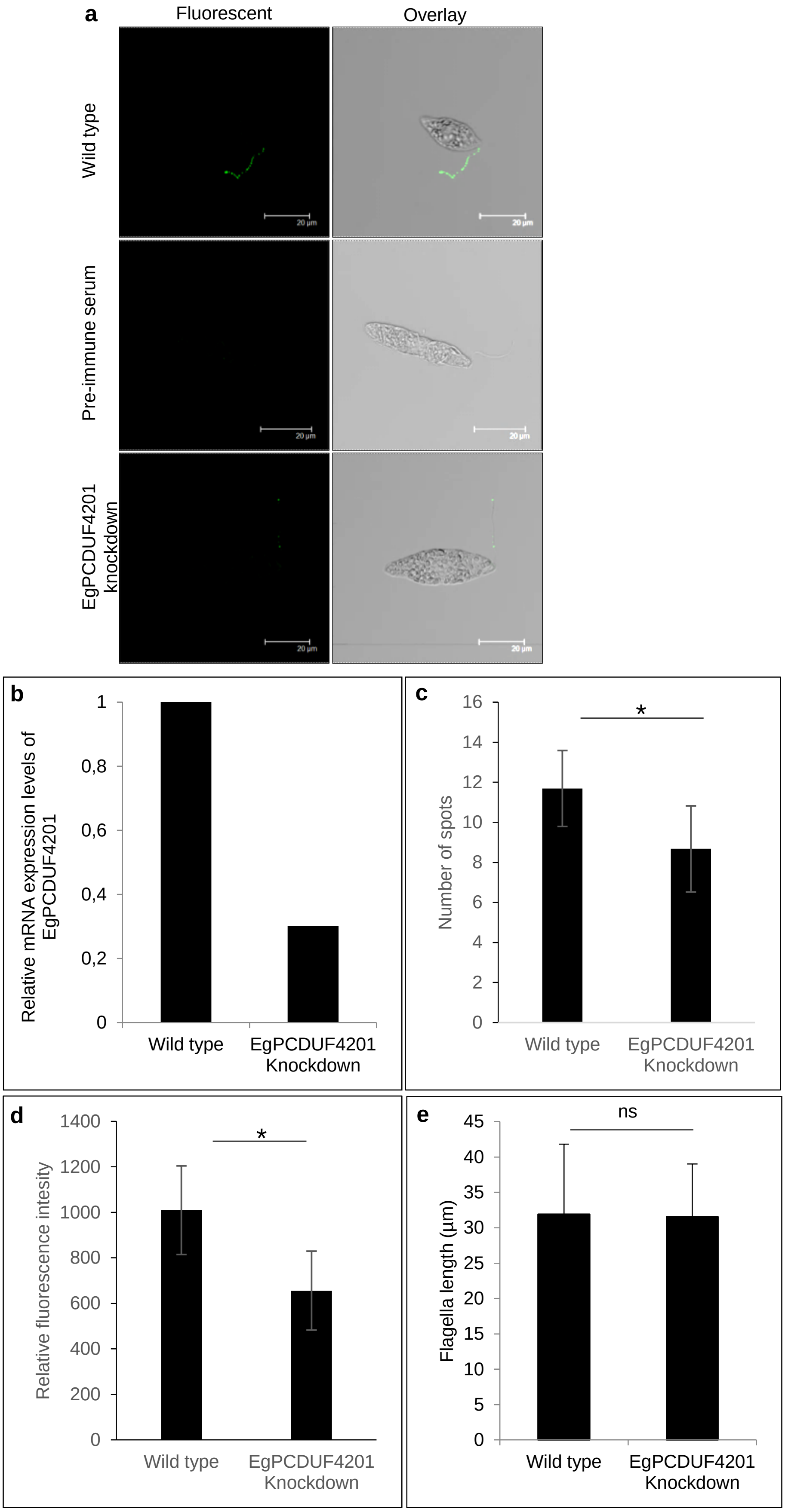

Supplementary Figure 5. Confirmation of EgPCDUF4201 localization. **a**. Indirect immuno-fluorescent assay with anti-EgPCDUF4201 serum (upper and lower panels), pre-immune serum (middle panel). **b**. Expression level of EgPCDUF4201 measured by real time PCR. **c**. Number of spots in the flagella of wild type cells and EgPCDUF4201 knockdown cells. **d**. Fluorescence intensity of the spots. **e**. Flagella length. N=50. * represents a significant difference of the student test.

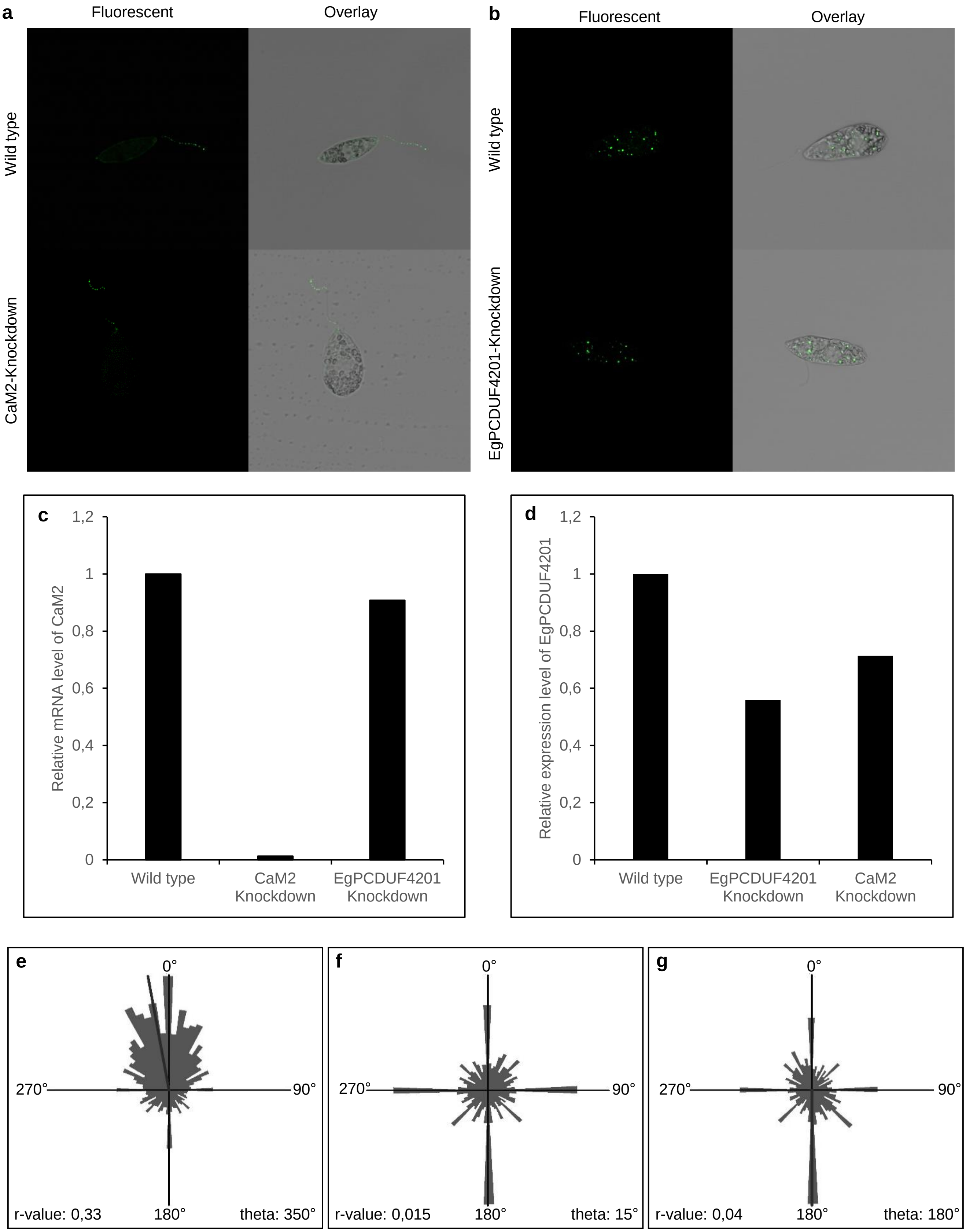

Supplemetary figure 6: EgPCDUF4201 and CaM2 localization and expression level in CaM2 and EgPCDUF4201 kockdown mutants. **a**. Localization of EgPCDUF4201 in CaM2 knockdown mutant. **b**. Localization of CaM2 in EgPCDUF4201 knockdown mutant. **c**. Expression level of CaM2 in EgPCDUF4201 knockdown mutant. **d**. Expression level of EgPCDUF4201 in CaM2 knockdown mutant. Representative histograms of the directional movements of wild type (**e**), CaM2 (**f**) and EgPCDUF4201 (**g**) knockdown mutants. The size of the angular sectors (represented in gray) reflects the number of cells moving in the corresponding direction.

	velocity [µm/s]
Control-C-ter-1	72,16262
Control-C-ter-2	62,81161
Average	67,487115
C-ter EgPCDUF4021 knockdown-1	66,98744
C-ter EgPCDUF4021 knockdown-2	64,51883
Average	65,753135
Control-N-ter-1	79,63938
Control-N-ter-2	80,96496
Average	80,30217
N-ter EgPCDUF4021 knockdown-1	76,46893
N-ter EgPCDUF4021 knockdown-2	75,06881
Average	75,76887

Supplementary Table 1. Velocity of the wild type and EgPCDUF4201 knockdown mutants.

Name	Restriction sites	Primer sequence (5'-3')
Primers for expression vectors		
pGBKT7-CaM2-for	NdeI	GTTTCATATGATGCCGGCCCTCACC
pGBKT7-CaM2- rev	BamHI	GTTTGGATCCTCATGCCCCCTTCAGGAT
pGBKT7-EgPCDUF4201-N-for	BamHI	GGATCCAATGCCGCCGCACGGCTTCAC
pGBKT7-EgPCDUF4201-N-rev	XhoI	CTCGAGCTAGTTGATGCGCACGTTGCGG
pGBKT7-EgPCDUF4201-C-for	BamHI	GGATCCATACATCAAGCTGCGCAACCGGA
pGBKT7-EgPCDUF4201-C-rev	XhoI	CTCGAGCTACGCTTGCGACGCCTCTGG
pGBKT7-EgPCDUF4201-full-length-for	BamHI	GGATCCAATGCCGCCGCACGGCTTCAC
pGBKT7-EgPCDUF4201-full-length-rev	XhoI	CTCGAGCTACGCTTGCGACGCCTCTGG
pGEX4T2-CaM2- for	BamHI	GGATCCATGAGCACGGTGACAAACAC
pGEX4T2-CaM2-rev	XhoI	GGATCCATGCCGGCCCTCAC
pGEX4T2-EgPCDUF4201-for	BamHI	GGATCCATGCCGCCGCACGGCTTCACG
pGEX4T2-EgPCDUF4201-rev	XhoI	CTCGAGCTACGCTTGCGACGCCTCTGGCAACTGTG
Primer used for 5'/3' RACE PCR		
EgPCDUF4201-RACE-rev1	NA	TTGCGCAGCTTGATGTAGTTGATG
EgPCDUF4201-RACE-rev2	NA	GTTGGTGGCGATCCACTTGAAGA
Cap for	NA	AAGACACTTTCTGAGTGTCTATTTTTTTTCG
Poly (A) rev	NA	TTTTTTTTTTTTTTTTTTTTTTTTTTTTTTVN
Primers used for qPCR/dsRNA		
CaM2-for	NA	GCAAGATCAGCTTCCAGGACTTCG
CaM2-rev	NA	TGCGAAAAGGTTGGGCAGGGGAAA
N-PCDUF4201-200-ds-for	NA	TCGCGAAGACGGAGGAG
N-PCDUF4201-200-ds-rev	NA	GCCTTTCATCTGCACCACC
C- PCDUF4201-200-ds-for	NA	CAGCGGAAATGGCGCGAGAGCCG
C- PCDUF4201-200-ds-rev	NA	GTTGTCCTGCAGCAGCCGCTCCC

Supplementary Table 2. All the listed primers were synthesized by a commercial vender eurofinsgenomics (Germany) (http://www.eurofinsgenomics.eu/). Restriction sites are underlined, capital N stand for N-terminus and C stands for C-terminus.

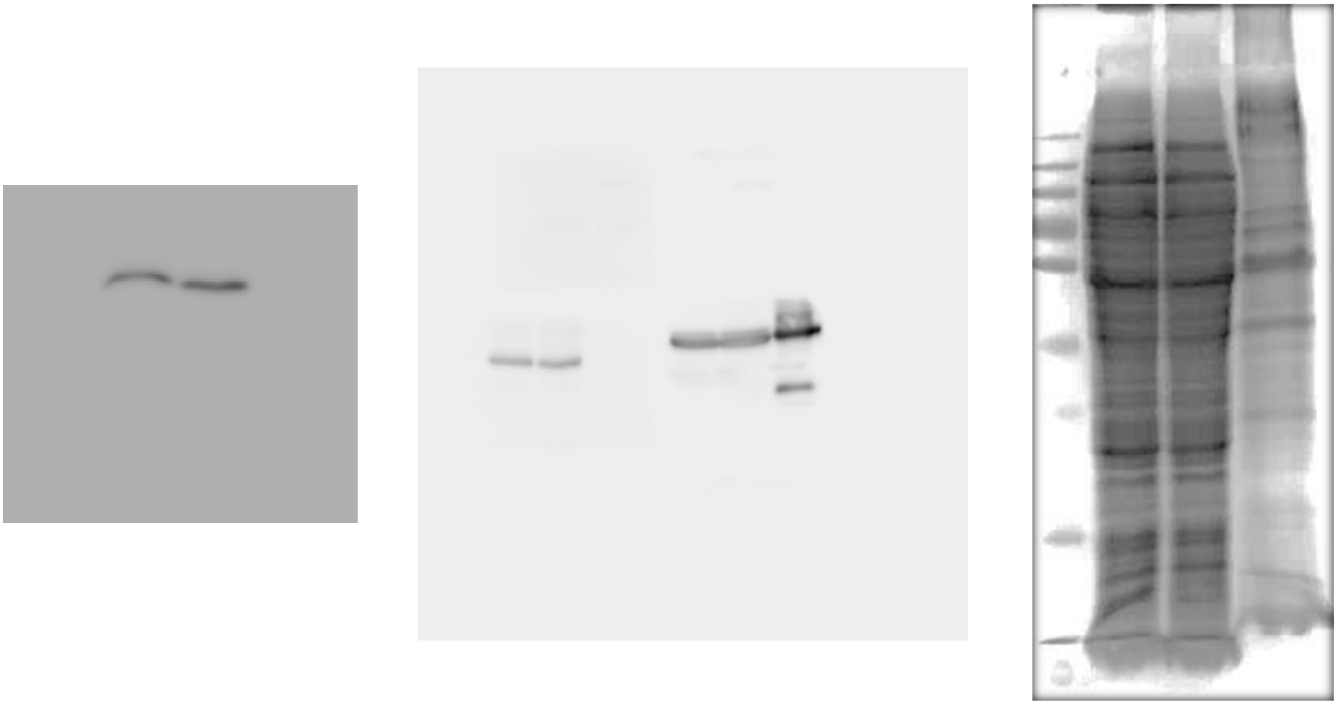

Full blots and gel of Figure 1

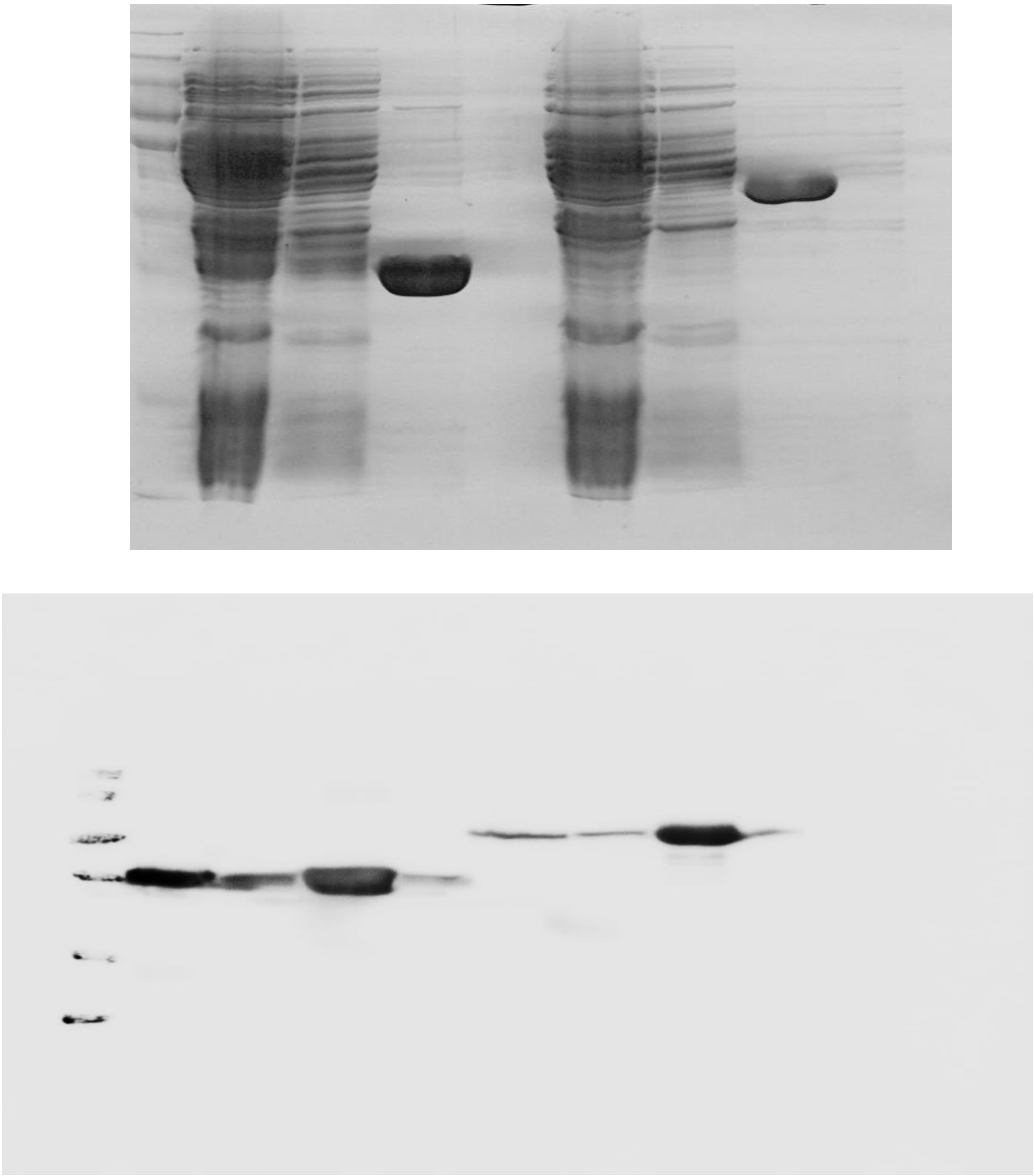

Full gel and blot of Figure 2

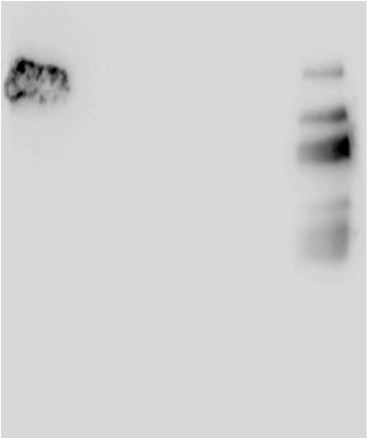

Full blot of Figure 3
